# Public involvement in suicide prevention: understanding and strengthening lay responses to distress

**DOI:** 10.1186/1471-2458-9-308

**Published:** 2009-08-23

**Authors:** Christabel Owens, Gareth Owen, Helen Lambert, Jenny Donovan, Judith Belam, Frances Rapport, Keith Lloyd

**Affiliations:** 1 Peninsula Medical School (Universities of Exeter & Plymouth), Wonford House, Dryden Road, Exeter, Devon, EX2 5AF, UK; 2 Department of Social Medicine, University of Bristol, Canynge Hall, Whatley Road, Bristol BS8 2PS, UK; 3 PAPYRUS prevention of young suicide, Burnley, Lancashire, UK; 4 School of Medicine, University of Swansea, Grove Building, Singleton Park, Swansea SA2 8PP, UK

## Abstract

**Background:**

The slogan "Suicide prevention is everyone's business" has been used in a number of campaigns worldwide in recent years, but most research into suicide prevention has focused on the role of medical professionals in identifying and managing risk. Little consideration has been given to the role that lay people can play in suicide prevention, or to the resources they need in order to do so.

The majority of people who take their own lives are not under the care of specialist mental health services, and around half have not had recent contact with their general practitioner. These individuals are not known to be 'at risk' and there is little or no opportunity for clinical intervention. Family members and friends may be the only ones to know that a person is troubled or distressed, and their capacity to recognise, assess and respond to that distress is therefore vitally important. This study aims to discover what the suicidal process looks like from the point of view of relatives and friends and to gain insight into the complex and difficult judgements that people have to make when trying to support a distressed individual.

**Methods/Design:**

The study uses qualitative methods to build up a detailed picture of 15–20 completed suicides, aged 18–34. Data are gathered by means of in-depth interviews with relatives, friends and others who knew the deceased well. In each case, as many informants as possible are sought using a purposive snowballing technique. Interviews focus on the family and social network of the deceased, the ways in which relatives and friends interpreted and responded to his/her distress, the potential for intervention that may have existed within the lay network and the knowledge, skills and other resources that would have helped members to support the distressed individual more effectively.

**Discussion:**

The study will inform interventions to promote public mental health awareness and will provide a basis on which to develop community-focussed suicide prevention strategies.

## Background

Improving the mental health of the population and preventing suicide are UK government health priorities. Around 5,000 people take their own lives in England every year, the majority being young, physically healthy adult males. Suicide is the commonest cause of death in men under the age of 35 [1]. These unnecessary deaths represent a significant loss to society and have far-reaching consequences for the health of others. Those bereaved by suicide often develop mental health problems themselves and lead permanently shattered lives as a consequence of their loss.

The slogan, "Suicide prevention is everybody's business", has been used in a number of campaigns around the world in recent years, including World Suicide Prevention Day in 2005 and International Suicide Awareness Week in 2006, and there is growing recognition of the need for whole community approaches to suicide prevention. The first National Suicide Prevention Strategy for England was launched in 2002 and identified the need for a broad-based approach that co-ordinates the efforts of public services, the private sector, voluntary groups and concerned lay individuals [1].

Despite this rhetoric, research has remained heavily focused on the role of healthcare professionals and on clinical prevention strategies, as well as on measures to restrict access to means. Little attention has been paid to the role of lay people in identifying and supporting distressed relatives and friends. It is therefore unclear what sort of contribution members of the public are able to make, and what resources they need in order to contribute effectively.

Understanding and maximising lay involvement is important because a significant proportion of those who take their own lives have no contact with health services, either primary or secondary, in the months prior to death [2]. Young people especially are known to be reluctant to use health services [3,4]. Non-users of services pose particular challenges in terms of identification of risk and opportunity for intervention. Their mental distress is invisible except to members of their social networks, whose capacity to recognise, assess and respond to that distress is therefore vitally important.

Studies in medical sociology have shown that relatives and friends play a key role in determining how health problems are defined and dealt with. They frequently act as lay consultants and providers of healthcare [5] and also sanction decisions to seek professional help [6-11]. Studies also show that lay people and doctors identify and evaluate 'symptoms' in very different ways, particularly in the contested field of mental disorder [6,12,13]. The relevance of this literature to suicide prevention has not previously been acknowledged. Virtually nothing is known about the family and social networks of those who take their own lives, about the ways in which those around them interpreted their distress, about whether they tried to intervene and in what ways, and about whether any further potential for intervention existed. Following in the Durkheimian tradition, most sociological research into suicide has confined itself to the macro level and has focused on the role of social variables in the aetiology of suicide [14-18].

A great deal of effort has gone into trying to understand the aetiology of suicide and to draw implications for prevention. This has been largely epidemiological in orientation and has concentrated on identifying risk factors through the use of quantitative methods. It has also tended to give precedence to psychiatric explanations. The voices of those who knew the deceased and witnessed events leading up to the suicide are largely absent from the literature, despite the fact that it has been common practice to interview them at length as part of the psychological autopsy procedure [19-21]. Psychological autopsy studies are designed to facilitate retrospective diagnosis of psychiatric disorders, rather than to elicit participants' perceptions of the circumstances surrounding the suicide. Thus, very few studies have paid attention to the meanings that bereaved relatives and friends attach to the act of suicide or to their views on how it might have been prevented. The potential of qualitative methods to illuminate this field is still largely unrecognised, even though research into lay understandings of health and illness is well established [22,23] and has been shown to be valuable in helping to understand and find solutions to public health problems [24-26].

In a first attempt to fill this gap, Owens et al transcribed the narrative accounts given by relatives during the course of a psychological autopsy study and analysed these using qualitative methods [27,28]. This approach not only provided insight into the ways in which those closest to the deceased accounted for the suicide, but also shed valuable light on help-seeking beliefs and behaviours. In many cases, family members had been aware of the individual's distress but appeared to have misjudged both its seriousness and its possible medical significance. Many had assumed that it was 'perfectly normal in the circumstances' (for example, after a relationship breakdown) and that the individual would 'get over it' in their own time. When dealing with mental distress, difficult and complex judgements have to be made within family and friendship networks about what is going on, and we know very little about how people make them and how they can be better equipped to do so. By studying in detail the social contexts within which particular suicides occurred and the ways in which relatives and friends interpreted and responded to distress, the present study will illuminate this neglected area.

The study aims to discover what the suicidal process looks like from the point of view of those close to the deceased; to learn more about the role that lay people might play in suicide prevention and the resources they need in order to do so, and to use this evidence to inform future interventions.

## Methods/Design

The study will employ a qualitative design involving in-depth interviews with multiple informants in order to build up a series of detailed case studies of completed suicides.

### Sample

In order to build on previous work [27-30], we are retaining our focus on individuals who had no contact with specialist mental health services during the 12 months prior to death. This population accounts for more than 75% of all suicides [31] but remains seriously under-researched. Members of this population pose the greatest challenge in terms of identification of risk and opportunity for intervention. They are effectively hidden within their communities and measures to identify and support them will depend heavily on lay involvement.

Maximum policy relevance will be achieved by focusing on the 18–34 age group. In the UK, the suicide rate among men under the age of 35 is well above that of the general population and causes particular concern as deaths among young people represent the greatest numbers of years of life lost. The study will include both sexes, in order to explore the comparative size and strength of their social networks, the flow of information around the network and other issues relating to prevention.

### Inclusion criteria

Individuals whose deaths were recorded as suicides by participating coroners, who died aged 18–34 and who had no contact with specialist mental health services during the 12 months prior to death.

### Exclusion criteria

Individuals whose deaths were not recorded as suicides; suicides under the age of 18 and aged 35 and over, and those in which the deceased had received care from specialist mental health services during the 12 months prior to death. Although it is common practice to treat open verdicts as suicides for research purposes, they are excluded from this study as it would be highly problematic to assume that relatives and friends believe the death to be a suicide and to question them as if it were. Even where deaths have been officially recorded as suicides, interviewers need to be sensitive to the possibility that some informants will not regard this as the correct verdict.

The study will produce detailed analyses of 15–20 cases, with as many informants as possible per case, in order to build up a full and nuanced picture of the deceased's social situation. For each case, we are aiming to interview a minimum of 3 people, including a family member, a close friend or peer and a work colleague, as well as the general practitioner (GP). Although not part of the lay network, GPs often have considerable insight into the deceased's family and social situation and may be able to identify ways in which lay people and health professionals could have worked together to support the distressed individual.

### Identification and recruitment of informants

Cases of completed suicide are being identified through Her Majesty's Coroners in London, South West England and South Wales. In the UK, in order to comply with the Data Protection Act 1998, researchers may be required to obtain honorary contracts in order to work in the offices of participating coroners and view personal data relating to the deceased.

**Figure 1:**
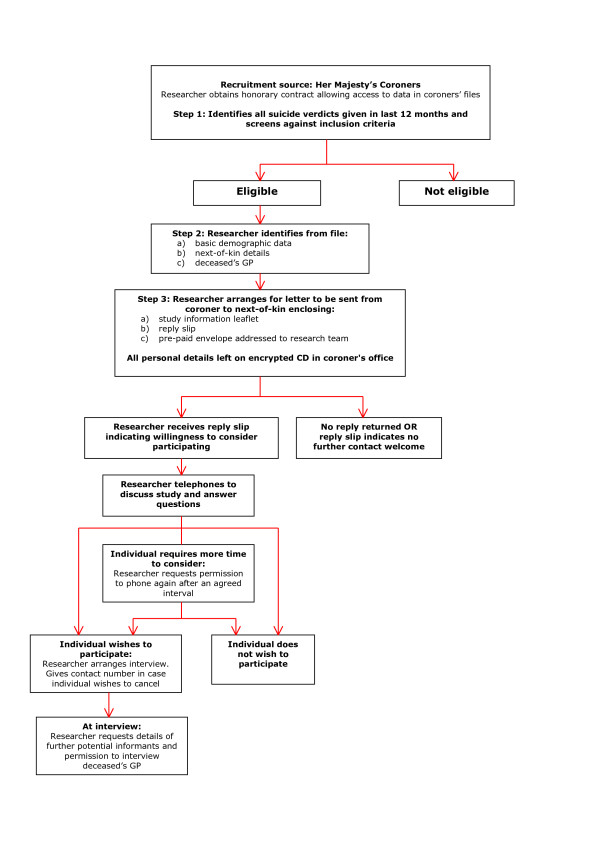
**Identification and recruitment of participants**.

The process of identifying and recruiting informants is outlined in Figure 1. From coroners' registers, researchers identify all suicide verdicts given within the last 12 months and assess them against the eligibility criteria. They then work consecutively through all eligible cases, examining the coroners' files and entering basic demographic data and salient details relating to family and social contacts directly into a database. Information to be gathered at this stage includes:

• name of deceased

• age

• sex

• ethnicity

• marital status

• occupation

• method of suicide

• next-of-kin name and address

• names of any other potential informants

• contact details of GP

This information is stored either on a password-protected desktop computer or on an encrypted CD and remains in the coroner's office until such time as all next-of-kin have indicated whether or not they are willing to participate in the study.

The next-of-kin of all eligible cases are approached in writing by the coroner who conducted the inquest. Decisions as to how soon after the inquest the letter should be sent are taken by coroners and their officers and are guided by their knowledge of each family's circumstances. This is likely to be between 3 and 12 months after completion of the inquest. Care is taken to avoid contacting the family around the time of the deceased's birthday and in the weeks leading up to the first anniversary of the death, in accordance with recommended procedure for psychological autopsy-type studies [20]. Enclosed with the letter of invitation is an information leaflet, a reply slip on which they can indicate whether or not they are willing to consider participating and a pre-paid envelope addressed to the research team.

If a next-of-kin does not wish to participate in the study, his/her personal details and those of all other potential informants, including the GP, are removed from the database. The deceased's case record is anonymised, but basic demographic details remain on the database to allow us to determine the extent to which cases included in the study are representative of the total population. Likewise, if an eligible case has no recorded next-of-kin and neither the information in the file nor conversations with the coroner's officer suggest any potential informants, the deceased's demographic data are entered but the case is logged as one that cannot be investigated. It is important to determine the proportion of such cases, since social isolation and lack of a personal network are known risk factors for suicide [32]. The database is fully anonymised before being removed from the coroner's office for analysis. The only personal details retained by the research team are those supplied by consenting next-of-kin.

On receipt of a reply slip from a next-of-kin indicating willingness to consider participating, a researcher makes contact by telephone, discusses the study and answers any questions. If the next-of-kin wishes to participate, the researcher makes arrangements for the interview to take place, ensuring that the informant knows how to contact the research team, should s/he change his/her mind and wish to cancel the appointment. If the informant requires more time to consider whether or not to participate, the researcher seeks permission to phone again after an agreed interval. Informants are asked to sign the formal consent form immediately prior to commencement of the interview.

The intention is to interview as many members of each deceased person's social network as possible, in order to gain different perspectives on the events leading up to the suicide and the social context in which it occurred. From previous work, it is clear that the accounts that family members give are motivated by a number of factors, including a desire to 'set the record straight' and to assign blame to other members of the deceased's social network [28]. In the present study, we purposively seek out those who may offer an alternative interpretation of events, both in pursuit of 'fair dealing' [33] and in order to build up a balanced understanding of the deceased's social situation and explore any ties or tensions that might have acted as barriers to awareness or impeded efforts to intervene.

As the first interview proceeds and the 'story' unfolds, key players will emerge. At the end of the interview, the researcher seeks the informant's permission to contact further members of the network and to interview the deceased's GP. A contact sheet is used to record the personal details of potential informants and to obtain the next-of-kin's consent to approach them. Nominated individuals are contacted by letter, telephone or e-mail, as recommended by the primary informant.

As each subsequent interview proceeds, the informant is asked if there are any further members of the social network who may have additional insights into the events leading up to the suicide and how it could have been prevented. This technique is commonly known as 'snowball sampling'. Snowball sampling is sometimes criticised on the grounds that it limits the pool to members of a specific network [34]. In certain types of study, however, that is precisely its strength. It has proved to be highly effective in studies of vulnerable, stigmatised, deviant or closed social groups, such as drug takers, homeless people or those affected by HIV/AIDS, as it enables in-depth knowledge of group beliefs and behaviours to be built up and gaps in knowledge to be identified and filled [35,36]. The researcher uses the trust that is built up in each interview in order to penetrate deeper into the social group and, by means of peer referral, obtain access to a range of perspectives that would otherwise remain hidden or inaccessible.

We continue snowballing until either no further informants are forthcoming or saturation is reached and no fresh information on the case is emerging. The use of progressive snowballing, or chain referral, ensures that our sample in each case is not restricted to those who are sanctioned by the next-of-kin. This is particularly important in the case of young people who may have been involved in drugs or other activities of which their parents were unaware.

### Interview methodology

The study uses an in-depth, narrative approach to interviewing that recognises and capitalises on participants' tendency to tell stories, particularly when talking about very painful experiences [28]. This approach relies on a single opening question that is designed to elicit an extended and uninterrupted narrative and to give the participant maximum control over the way in which material is organised and presented [37-39]. The rationale for adopting this approach is both scientific and ethical. By leaving participants free to decide what to include and how to structure their story, rather than asking predefined questions and imposing a structure, it provides far greater insight into the meanings and relative significance that particular events have for them. From an ethical point of view, given the very distressing nature of the subject matter, it also allows the participant to feel more relaxed and secure, knowing that they can disclose information at their own pace [37,40,41].

In this study, the participant is asked: 'Please tell me, in your own time, about [the deceased], about your relationship with him/her and about what happened in the period leading up to his/her death.' The interviewer then takes a back seat and assumes the role of 'active listener', noting down points to follow up later but not interrupting. Once the free flow of narrative has come to a halt, the interviewer asks follow-up questions based on the narrative itself, picking up any points within the story that require clarification or elaboration and also offering 'pertinent ways of conceptualizing issues and making connections' for the participant to consider [42]. This second part of the interview is very much a dialogue, in which interviewer and interviewee work together to construct an account of the suicide and the context in which it occurred that addresses the needs of the study. A topic guide is used as a prompt to the interviewer, in order to ensure that all areas of interest are covered.

Finally, the participant is encouraged to construct a visual representation of the deceased's social network, using pebbles, sticky notes and coloured pens to indicate members of the network, their relative proximity to the deceased, the quality of their relationships with each other and the paths by which information travelled around the network. Interviews are audio-taped and transcribed verbatim.

### Data analysis

Data analysis begins as soon as the first interview has been conducted and occurs alongside data collection, to allow the interviews to become progressively focused and emerging hypotheses to be tested and refined.

The data lend themselves to three types of analysis: narrative analysis of individual accounts; detailed case studies, built up using the multiple accounts relating to each case, and general cross-case comparisons based on both thematic and narrative analysis. We start with the analysis of individual narratives. On the basis of an initial reading of a number of transcripts by all team members, key themes are identified and a coding frame is developed, which is then applied systematically to all transcripts. As each transcript is coded, the textual material relating to each theme is summarised and recorded, together with line references, illustrative quotations and interpretive comments, on an individual narrative proforma. The proforma allows the overall integrity of each separate account to be preserved and provides the basis for narrative analysis, with its emphasis on structure, plot and performance, rather than on content alone [43]. Participants are offered the opportunity to peruse their own interview transcript and the narrative proforma, in order to validate or contest our interpretation of their story and to offer any further thoughts that have occurred to them since the interview.

We then 'unpack' each theme, breaking the material down further into subtopics and smaller categories and using thematic charts to provide a 'viewing platform' [44] from which to compare different accounts, both within and across cases. The charts are used to produce descriptive accounts of each theme, identify deviant accounts and generate tentative hypotheses for testing against the full data set.

Individual case studies are built up using all the accounts relating to a case, identifying discrepancies and areas of agreement between informants and relating these to their position within the social network. The visual 'map' of the social network that is constructed with participants during the interview is used to contextualise individual accounts. Using a timeline approach, we aim to build up a detailed picture of:

• what signs of distress each member of the network picked up;

• how they interpreted these, both at the time and in retrospect;

• what actions they took, including communicating concerns to other network members and seeking help from outside agencies;

• what barriers to awareness and intervention operated;

• what further potential for intervention may have existed;

• what knowledge and skills might have enabled them to manage the situation differently and possibly prevent a fatal outcome.

The intention is to gain insight into the suicidal process, as it unfolded within a particular micro-social context and as witnessed by social network members. We are particularly interested in the way in which the deceased interacted with and managed his/her social network and the way in which personal relationships may have impacted on the ability to recognise and respond to risk of suicide.

We then look across cases, using the emergent themes to transcend the particular, compare and contrast cases of suicide and develop generalisations that represent the total data set [45]. These will provide the basis for the development of hypotheses and interventions for testing in future studies.

## Discussion

This micro-analytic approach to the suicidal process and the social networks within which it unfolded is entirely novel. Durkheim argued that suicide is not just an individual act and cannot be explained by individual attributes alone, but that the characteristics of society affect the probability of suicide in its members and thus determine suicide rates, with the key determinant being the extent to which individuals are integrated or bonded into social groups [14]. More than a hundred years on, few studies have examined closely the social networks of those who take their own lives, the nature of the bonds that unite individuals and the ways in which these may promote or constrain both suicidal behaviour and attempts to intervene.

Large-scale epidemiological and psychological autopsy studies have considered the size of individuals' interpersonal networks, using the number of relationships and frequency of social contacts as variables, and suggest a straightforward association between social isolation or a lack of social ties and suicide [32]. As Maris comments, 'Generally, the more "significant others" the lower the suicide rate' [46]; 'having more people around promotes suicide prevention and intervention' [47]. This quantitative approach does not help us to understand why those with apparently strong and supportive social networks still take their own lives and why those who love and wish to protect them are often powerless to intervene [28].

Our in-depth qualitative approach will allow us to look closely at these issues and to understand how best to equip members of the public to recognise and respond to suicide risk in those around them. It will provide a firm foundation on which to base interventions to raise mental health awareness and promote a community-wide approach to suicide prevention. Study participants and members of relevant voluntary organisations will be invited to work with the research team on future development activities.

### Ethical issues

Interviewing those who have suffered a traumatic bereavement is a delicate procedure requiring the utmost sensitivity, respect for the integrity of the deceased and concern for the health of the informant. However, there is evidence to suggest that research interviews following a suicide, if conducted sensitively, can be therapeutic [20,48,49]. Because of the stigma and taboos surrounding suicide, those who are bereaved in this way have limited opportunities to talk openly about it and are often grateful for the opportunity to do so.

It is quite possible that informants will become upset, agitated and even angry during the course of the interview. Feelings of guilt, shame and anger are common among those bereaved by suicide [50,51]. They often blame another person for the death and it is not uncommon for informants to paint highly defamatory pictures of other members of the family or social network, whom we may wish to interview [28]. Such situations require skilful and sensitive management. Some researchers have recommended using different interviewers to interview different members of a family or social network in order to ensure equity and to reduce the possibility of researchers being drawn into family conflicts [41]. Special care must be taken to ensure that relatives and friends of the deceased are not made to feel that they were responsible for the death or should have done more to prevent it.

At the close of the interview, the interviewer should not leave before ensuring that the participant is in a stable emotional state and has adequate personal support. If the interviewer has any concerns about the participant's mental state, s/he should offer to contact the participant's GP. This is particularly important if the participant is expressing any suicidal ideation or intent. All participants will be given an information pack containing details of bereavement support organisations and relevant reading material. A post-interview personalised letter or email will be sent, thanking the participant for their time, enquiring how they have been feeling and inviting them to get in touch should any issue arise in relation to the interview. Arrangements will also be made with appropriate voluntary organisations for post-interview support to be available to study participants. Interviewing the bereaved on such a sensitive topic places great emotional strain on interviewers and arrangements will be made for regular debriefing and supervision [20,48,52].

Ethical approval for the study was granted by London NHS Multi-centre Research Ethics Committee.

## Competing interests

The authors declare that they have no competing interests.

## Authors' contributions

CO, HL, KL and JD conceived the study. All authors contributed to study design and protocol development and have read and approved the manuscript.

## Pre-publication history

The pre-publication history for this paper can be accessed here:


